# Real-Time Integration of Bronchoscope Video and 3D Airway Models on Apple Vision Pro: Phantom Feasibility Study

**DOI:** 10.2196/103880

**Published:** 2026-10-30

**Authors:** Shotaro Okachi, Tomoya Horiguchi, Yasuhiro Goto, Michitaka Fujiwara, Kazuyoshi Imaizumi, Naozumi Hashimoto

**Affiliations:** 1 Department of Respiratory Medicine, Fujita Health University School of Medicine, 1-98 Dengakugakubo, Kutsukake-cho, Toyoake, Aichi, 470-1192, Japan, 81 562 93 2111; 2 Medical xR Center, Nagoya University Graduate School of Medicine, Nagoya, Aichi, Japan

**Keywords:** Apple Vision Pro, spatial computing, extended reality, mixed reality, bronchoscopy, virtual bronchoscopy, network device interface, phantom simulation, feasibility study

## Abstract

This single-operator phantom study demonstrates the technical feasibility of colocating a live bronchoscope video stream with patient-specific 3D airway models on Apple Vision Pro using a native Network Device Interface pipeline, without establishing comparative or clinical benefit.

## Introduction

Bronchoscopic biopsy of peripheral pulmonary lesions remains technically demanding because the operator must mentally translate 2D computed tomography (CT) images into the 3D bronchial route.

Mixed-reality (MR) head-mounted displays ease this load by keeping a positionable 3D reference in view during the procedure. Our group has used optical see-through MR (using HoloLens 2 by Microsoft and Holoeyes MD by Holoeyes Inc) for intraprocedural 3D holographic virtual bronchoscopy [1,2]. Separately, without this platform, we have shown the feasibility of Apple Vision Pro (AVP) for simulation-based bronchoscopy training [3].

We developed a prototype visionOS application that ingests the bronchoscope feed via the Network Device Interface (NDI; Vizrt Group) and renders it as a plane colocated with the patient-specific 3D airway model. We report a single-operator phantom feasibility study as a proof of concept.

## Methods

### Ethical Considerations

Existing clinical CT images of patients at Fujita Health University Hospital were used retrospectively and anonymized to generate the models. The institutional review board of Fujita Health University approved the study (HM25-343) and waived written informed consent, with an opt-out procedure, in accordance with the Japanese Ethical Guidelines for Medical and Biological Research Involving Human Subjects. No procedures were performed on patients or volunteers.

### Application

We established a native visionOS 2 application (Swift, SwiftUI, RealityKit) that receives a live video stream over NDI through a custom Swift bridge to the NDI SDK for Apple Silicon and renders it colocated with patient-specific 3D airway models. Two STL meshes per case—the airway tree (pink) and the target nodule (green)—are loaded at runtime. Using ARKit hand tracking, the video plane and each model can be independently repositioned, rescaled, or hidden. No third-party MR platform was used.

### Datasets

Ten patient-derived datasets, each a different bronchopulmonary segment, were generated from thin-slice chest CT images (0.5‐1.0 mm) on a workstation (Ziostation REVORAS, Ziosoft) using the same pipeline as our previous MR virtual bronchoscopy work [1,2].

### Bench Setup and Protocol

An aScope 5 Broncho bronchoscope (4.2/2.2; Ambu A/S) was advanced in a Koken LM-099 airway phantom (Koken). Its image was passed by HDMI (High-Definition Multimedia Interface) cord from the Ambu monitor to a dedicated computer running the Ambu Broncho Simulator (version 2; Ambu A/S), a platform that labels bronchial segments. The simulator output was captured using a USB HDMI capture card (CM489; UGREEN) on a MacBook Air (M2; Apple Inc) and sent to AVP through OBS Studio with the DistroAV plug-in via NDI over a 5-GHz personal hotspot (iPhone 16; Apple Inc). The experimental setup is shown in Figure 1A and the operator’s view is shown in Figure 1B and Multimedia Appendix 1.

**Figure 1.**
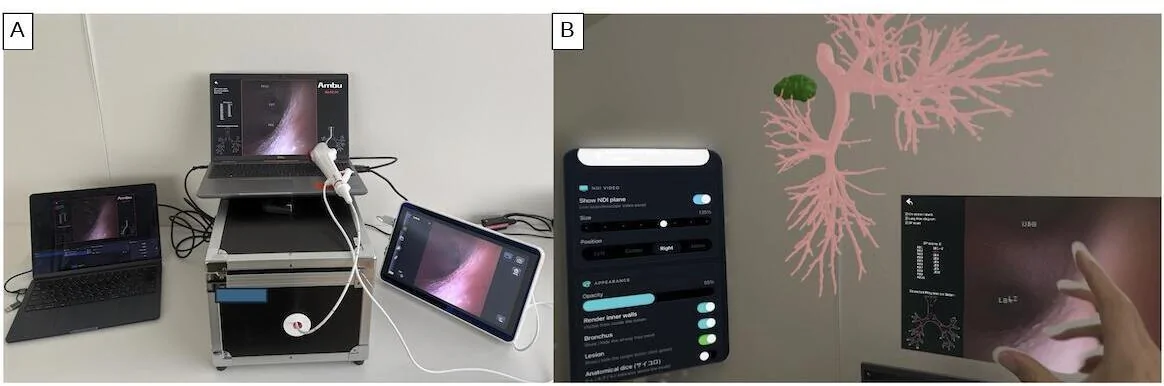
Experimental setup and operator’s view. (A) Hardware configuration: a flexible bronchoscope (aScope 5 Broncho 4.2/2.2) was inserted into a Koken LM-099 airway phantom (center), with its handle resting on top. The bronchoscope image is shown on the Ambu monitor (right), passed by HDMI cable to a dedicated computer running the Ambu Broncho Simulator AI software (on the phantom case, center) and then relayed through a USB capture card to a MacBook Air (left), which sends the stream to AVP via NDI. (B) The operator’s first-person view via AVP during a trial: the live bronchoscope video panel (right) is colocated with the patient-specific 3D airway model (pink) and the application control panel (left); the target nodule is shown in green. The 10 target segments were L1+2, L3, L4, L6, L8, L9, R2, R3, R5, and R7. AVP: Apple Vision Pro; HDMI: High-Definition Multimedia Interface; NDI: Network Device Interface.

A single board-certified bronchoscopist (SO, 20 years of experience) performed 10 trials in one session in random segment order. For each trial, the corresponding dataset was loaded, and the operator advanced the bronchoscope to the target segmental orifice while viewing the NDI video plane and the 3D model on the AVP. The operator’s view is shown in Multimedia Appendix 1.

### Outcomes

Primary outcomes were per-trial time-to-target and reach success (binary). Time-to-target was read from the simulator’s built-in timer, which started when the scope passed the carina and was recorded when the target segmental label changed color. Secondary outcomes were operator-reported Simulator Sickness Questionnaire (SSQ) scores before and after the session [4] and a free-text log of instability. Transport latency was measured separately (Multimedia Appendix 2). No statistical inference was performed.

## Results

All 10 target segments were reached, for a per-trial reach success of 10/10 (100%). The mean time-to-target was 37.5 (SD 5.0; range: 30‐44) seconds.

The integrated workspace remained operable throughout the session, which was completed without removing the headset. A single transient video freeze occurred during one trial (R5), from which the stream recovered immediately; its cause was subsequently identified as unbounded frame queuing in the NDI receiver and corrected. No loss of control, image corruption, or other instability occurred in the remaining trials. Latency was not measured during the trials; in bench measurements made 3 months later, on a subsequently modified build, latency of the NDI path relative to pass-through was 250 (median; IQR 232‐261) milliseconds, approximately 261 milliseconds from sensor to display (Multimedia Appendix 2).

Total SSQ severity was 0 before and 7.5 after the session. The increase was confined to the oculomotor domain (general fatigue and eyestrain, each rated slight). No nausea or disorientation was reported, and no adverse events occurred.

## Discussion

To the best of our knowledge, this is the first report to colocate a live bronchoscope video stream—ingested through a native NDI-based pipeline, without a third-party MR platform—and a patient-specific 3D airway model in a single immersive scene on AVP.

Our work shares a live-video approach with recent surgical applications of AVP [5,6] but applies it to peripheral bronchoscopy and colocates the endoscopic image with the patient-specific 3D airway model in one scene rather than beside separate 2D windows.

This study included no comparator, neither conventional bronchoscopy nor the 3D model without colocated video, and therefore cannot determine whether the integrated display confers any benefit over existing approaches; all findings are descriptive. The 10/10 reach success in a static phantom by a single experienced bronchoscopist is near-ceiling by design, cannot be attributed to the system, and is not evidence of efficacy. Target reach was assessed only to the segmental bronchus, the level the simulator labels. Workload was not assessed beyond the SSQ. The measured latency is not negligible; it was obtained 3 months after the trials on a modified build, at a single short range, and excluded the Ambu Broncho Simulator stage present during the trials, so it underestimates that configuration. A dedicated low-latency network and further reduction are prerequisites for clinical use. If the stream is interrupted, the video plane stalls while the locally rendered 3D model and the bronchoscope’s own monitor remain available, though this fallback was untested.

Future studies should include a controlled comparison against conventional bronchoscopy and against the 3D model without colocated video, a multioperator study with blinded timing, and an approved first-in-human case with a coregistered 3D model.

## Supplementary material

10.2196/103880 Multimedia Appendix 1 Representative first-person recording of the operator’s view through Apple Vision Pro during use of the integrated display, showing the live bronchoscope video colocated with the patient-specific 3D airway model. This is an illustrative demonstration recorded separately from the timed trials.

10.2196/103880 Multimedia Appendix 2 Latency measurement: rationale, method, conditions, results, and limitations.

## Abbreviations

AVP: Apple Vision Pro
CT: computed tomography
HDMI: High-Definition Multimedia Interface
MR: mixed-reality
NDI: Network Device Interface
SSQ: Simulator Sickness Questionnaire
