# Supplementary material for: Real-Time Integration of Bronchoscope Video and 3D Airway Models on Apple Vision Pro: Phantom Feasibility Study
Source: JMIR XR Spat Comput. 2026 Oct 30;3:e103880. doi: 10.2196/103880 (PMC13722140; doi:10.2196/103880)
Supplement: Supplementary file 1 [file xr-v3-e103880-s002.docx]

**Multimedia Appendix 2. Latency Measurement**

**Rationale and definition.**

Sensor-to-display latency is a critical parameter for any real-time intraprocedural display. It is defined here as the interval between light entering the bronchoscope sensor and the corresponding image being emitted by the Apple Vision Pro (AVP) display. The visionOS internal display cannot be filmed externally, because it powers off when removed from the head, and AirPlay mirroring or external screen capture would themselves add delay and invalidate the measurement. Latency was therefore measured from on-headset screen recordings using a self-cancelling difference method.

**Method.**

A millisecond clock displayed on a monitor was viewed simultaneously in two ways: (1) directly through the device's video passthrough, and (2) after traversing the full transport pipeline (bronchoscope, HDMI, USB capture, OBS Studio with the DistroAV NDI plug-in, Wi-Fi, AVP NDI decode, RealityKit texture, compositor, display) on the co-located NDI panel. Both clocks are composited into the same recorded frame at the same instant, so the recording's own capture delay affects them equally and cancels in their difference. The difference therefore equals the latency of the NDI path relative to video passthrough. Adding the see-through (photon-to-photon) latency of the headset's own video passthrough approximates the absolute sensor-to-display latency; the manufacturer states this as 12 ms, and independent bench measurement has put it at approximately 11 ms. Values were read frame by frame and are reported as median and interquartile range (IQR), because network latency is right-skewed. The IQR describes the spread of the individual readings and should not be read as the accuracy of the estimate, which is bounded by the per-frame reading uncertainty given below.

**Measurement conditions.**

NDI stream: full NDI, 1920 x 1080, estimated 100–150 Mbps. Wireless link: the same personal hotspot used for the timed trials (iPhone 16, Maximize Compatibility disabled, so the hotspot operated on 5 GHz); sender-to-receiver range approximately 1 m. A dedicated link of this kind was necessary rather than a matter of convenience. NDI discovers devices by multicast and then streams directly between them on the same subnet, and the institutional wireless network segments and isolates clients so that this traffic does not pass; in our setting, NDI sources on the institutional network were discoverable but did not become connectable, and a dedicated link was therefore used. A full NDI stream at this bitrate would in any case be inappropriate on a shared clinical network. This is the practical reason why a dedicated wireless network is stated in the Discussion as a prerequisite for clinical use. Screen recording: 30 frames per second. Read resolution is limited by the 33-ms recording interval and, more severely, by motion blur of the fastest-changing digits: because the millisecond counter advances many times within one exposure, the last one or two digits are doubled in every frame. The counter can therefore be read only to its hundreds-of-milliseconds digit on the clocks viewed through the bronchoscope, and to its tens-of-milliseconds digit on the directly rendered source clock. Individual frames are accordingly read to within approximately 50 ms.

**Results.**

| **Measure** | **Median** | **IQR** | **Range** | **Frames** | **Recordings** |
| --- | --- | --- | --- | --- | --- |
| Latency of the NDI path relative to passthrough | 250 ms | 232–261 ms | 217–273 ms | 15 | 2 |

Timing of the measurement. The ten timed trials were performed on 8 June 2026; this latency measurement was made on 6 September 2026. In the interval the application was modified: while preparing the measurement we identified and corrected two defects in the NDI receiver, namely unbounded queuing of decoded frames on the main actor, which also caused the transient video freeze observed in trial R5, and an unnecessary per-frame CPU pass over the full 2-megapixel image. The value above therefore characterises the corrected build and not the build used for the trials, which was never measured.

**Limitations of this measurement.**

First, latency was not measured during the timed trials, and the build that ran them was not measured; the value above was obtained on the bench three months later on a modified build, as described above. Second, although the measurements used the same 5-GHz hotspot as the timed trials, they were made at a single short sender-to-receiver range and do not characterise performance at longer range or on a congested link. Third, the Ambu Broncho Simulator stage present in the trial chain was unavailable for this measurement and was therefore not in the measured chain. The reported value represents the transport latency of the NDI-based integration alone and, because the omitted stage can only add delay, it underestimates the latency of the configuration actually used in the timed trials. Fourth, the samples are frames read from two continuous recordings and are not independent events; the repeated identical values in the corrected condition reflect the read resolution stated above. The per-frame reading uncertainty of approximately 50 ms is a property of the apparatus and is not reduced by reading more frames, although the median over many frames is less affected than any single reading. Fifth, the passthrough latency used to convert the relative figure to an absolute one is taken from published figures for this headset and was not measured in our setup.
